# A low-cost, battery-powered acoustic trap for surveilling male *Aedes aegypti* during rear-and-release operations

**DOI:** 10.1371/journal.pone.0201709

**Published:** 2018-08-02

**Authors:** Brian J. Johnson, Barukh B. Rohde, Nicholas Zeak, Kyran M. Staunton, Tim Prachar, Scott A. Ritchie

**Affiliations:** 1 College of Public Health, Medical and Veterinary Sciences, James Cook University, Cairns, Queensland, Australia; 2 Australian Institute of Tropical Health and Medicine, James Cook University, Cairns, Queensland, Australia; 3 Department of Electrical & Computer Engineering, University of Florida, Gainesville, Florida, United States of America; 4 Verily Life Sciences, South San Francisco, California, United States of America; University of California Davis, UNITED STATES

## Abstract

The *Aedes aegypti* mosquito is a primary vector of several serious arboviruses throughout the world and is therefore of great concern to many public health organizations. With vector control methodology pivoting towards rearing and releasing large numbers of genetically modified, sterilized, or *Wolbachia-*infected male mosquitoes to control vector populations, economical surveillance methods for release tracking becomes increasingly necessary. Previous work has identified that male *Ae*. *aegypti* are attracted to female wingbeat frequencies and can be captured through artificial playback of these frequencies, but the tested systems are cost-prohibitive for wide-scale monitoring. Thus, we have developed a simple, low-cost, battery-powered, microcontroller-based sound lure which mimics the wingbeat frequency of female *Ae*. *aegypti*, thereby attracting males. We then tested the efficacy of this lure in combination with a passive (non-powered) gravid *Aedes* trap (GAT) against the current gold-standard, the Biogents Sentinel (BGS) trap, which requires main power (household power) and costs several times what the GAT does. Capture rates of male *Ae*. *aegypti* in sound-baited GATs (Sound-GATs) in these field tests were comparable to that of the BGS with no inhibitory effects of sound playback on female capture. We conclude that the Sound-GAT is an effective replacement of the costly BGS for surveillance of male *Ae*. *aegypti* mosquitoes, particularly in the developing countries where funding is limited, and has the potential to be adapted to target males of other medically important species.

## Introduction

The mosquito *Aedes aegypti* is the primary vector of dengue, chikungunya, Zika, and yellow fever viruses, causing millions of cases of these mosquito-borne illnesses each year [1–3]. Traditional wide-scale application of insecticides has largely failed to control dengue [4], particularly as the public’s tolerance for mass spraying wanes and resistance to commonly-used pesticides such as deltamethrin and pyrethroids becomes common in *Ae*. *aegypti* [5, 6]. Instead, “rear and release” methods of vector control [7], involving the rearing and release of effectively sterile or genetically modified mosquitoes to suppress vector populations or virus transmission, have become increasingly popular [8–10]. Programs focusing on population suppression through the release of *Wolbachia-*infected (e.g. Verily Debug Project [11] and MosquitoMate [12]), genetically modified (Oxitec [13]) or irradiated males (IAEA [9, 14]), require the ability to monitor wild and released males to effectively manage their releases. Unfortunately, because most commercially available traps are designed to capture either host-seeking or oviposition-ready females, accurate estimates of male populations are unattainable with existing tools.

The majority of male-based suppression programs rely on the Biogents Sentinel trap (BGS; Regensburg, Germany) to satisfy their female and male *Aedes* surveillance needs [10–13, 15]. Although the BGS is rightly considered the current gold standard for urban *Aedes* capture [16], it is expensive (US$197, Bioquip.com) and relies on a power-hungry electric fan. In addition to high costs, its electrical requirements impose significant surveillance and logistical limitations, the largest being that it requires access to mains power (household) or large 12 V batteries. Large batteries or use of solar panels for long-term surveillance also present a large risk of device theft or tampering, additionally fan motors can fail necessitating extra expenses replacing them [17, 18]. These short-comings result in a lack of fine-scale surveillance required to obtain accurate estimates of male population sizes. To overcome these failings, researchers recently demonstrated the successful exploitation of the attraction of male *Ae*. *aegypti* to female flight tones to capture males in non-mechanical, passive (no powered components) gravid *Aedes* traps (GAT) [19]. The success of this method is based on the knowledge that male *Ae*. *aegypti*, like most mosquito species, use auditory sensory organs to detect and locate female mosquitoes by recognizing the female’s unique flight tone [20–22]. This attraction can be exploited to capture males through artificial playback of conspecific tones, but typically only after initiation of male swarming behaviour around visual ‘swarm markers’. In the case of urban *Aedes* these markers are commonly bloodmeal hosts or dark objects attractive to females [23–25]. Though the GAT on its own does not capture a meaningful number of males as it is designed to capture egg-laying females, it does act as an effective visual swarm marker helping concentrate males and increasing efficacy of sound assisted capture. Although sound-baited GATs (Sound-GAT) reduce the power requirements and costs of male surveillance, the tested sound lures were relatively expensive (ca. $20), had a short battery life (<48 h), and did not allow for individual programming to optimise frequency playback for individual surveillance needs. Here, we report the development of a low-cost, low-power microcontroller-based acoustic lure to address the power and cost restrictions of previously tested sound lures. The efficacy of this lure, placed in a GAT to create a more affordable and long-lasting male monitoring tool, is assessed against BGS traps in field trials in Cairns, Australia.

## Methods

### Device construction

**Fig 1:**
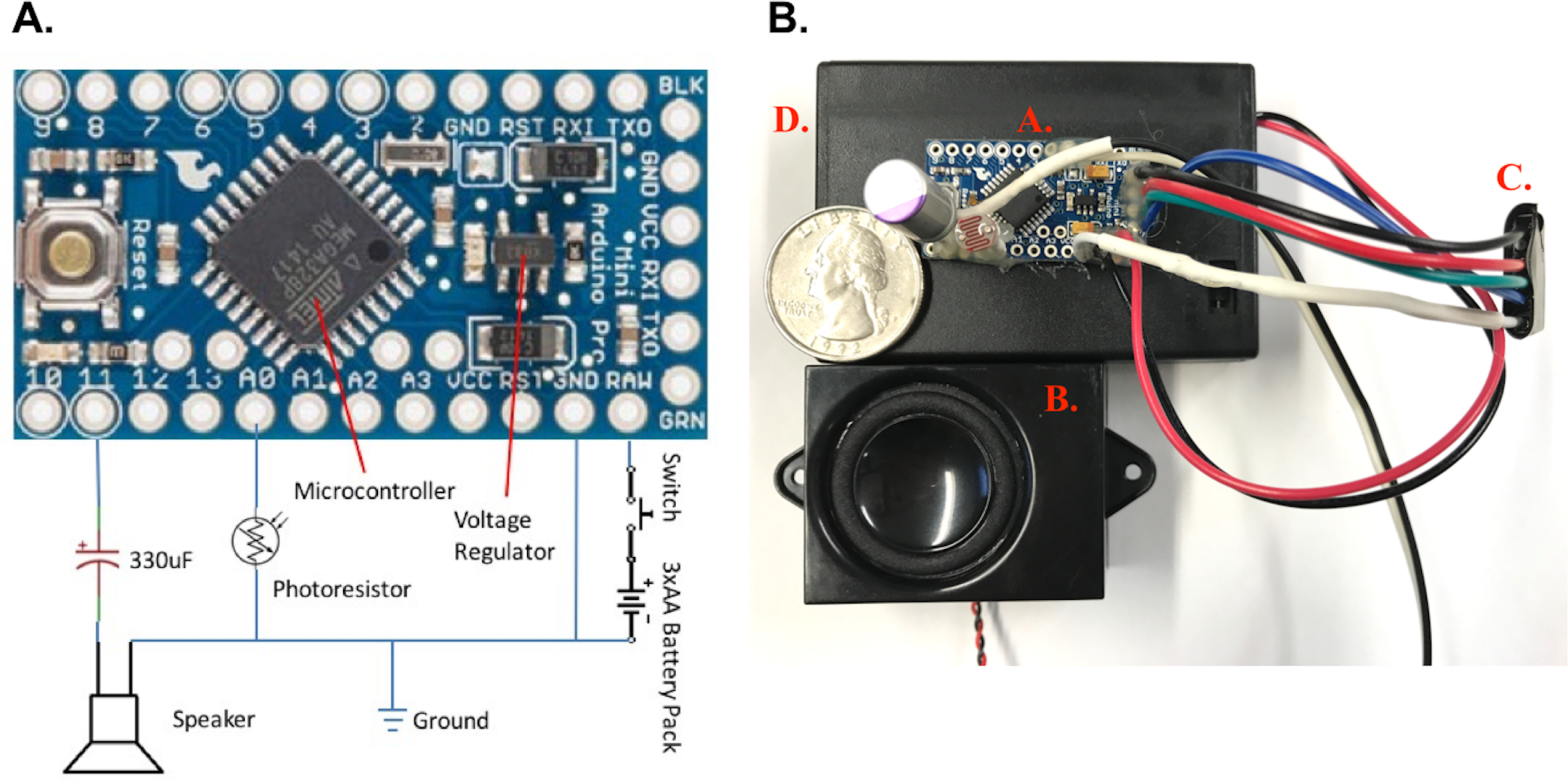
Diagram of sound lure. **(A)** Detailed schematic of the Arduino-based sound lure (Pro Mini 3.3 V board). The lure is programmed to produce a pulse-width-modulated 484 Hz sinusoidal-approximating signal through pin 11, from which it would travel through a DC-blocking capacitor to a speaker. The device is powered via a battery pack (SBH331AS, Memory Protection Devices Inc, Farmingdale, NY), containing three AA batteries and an on/off switch. **(B)** Assembled sound lure with a United States quarter dollar (diameter 2.54 cm) on top of battery box for size comparison. The components are: (A) assembled Arduino board, (B) speaker, (C) wired TTL serial adapter to connect to FTDI USB to TTL serial adapter to enable programming of board (Note: this component can be removed after programming if desired) and (D) battery pack.

The device (Fig 1, Table 1), powered by three AA batteries, is based on an Arduino Pro Mini 3.3 V PCBA platform (SparkFun Electronics, Colorado, USA). The Arduino Pro Mini schematic is available at https://cdn.sparkfun.com/datasheets/Dev/Arduino/Boards/Arduino-Pro-Mini-v14.pdf (accessed May 25, 2017). The microcontroller was programmed to output a pulse imitating a 484 Hz sinusoid, to save the cost of a digital-to-analog converter while limiting the harmonics produced by a square wave [26]. A tone of 484 Hz was chosen based on its demonstrated attraction to male *Ae*. *aegypti* in previous reports [19]. This waveform was fed through a DC blocking capacitor (330 nF, 10 V) to a magnetic speaker (ASE04508MR-LW150-R, PUI Audio Inc, Dayton, OH). A photocell (PDV-P8103, Luna Optoelectronics, Luna Inc, Roanoke, VA) was used to detect ambient light. The device was programmed to be active only during the day to conserve power and focus on the period when *Ae*. *aegypti* are most active [27, 28]. A detailed summary of construction costs and components are summarized in Table 1. When set in the GAT (Biogents AG; Regensburg, Germany), the device was placed inside the collection chamber on top of the insect screening, the detailed specifications of which are found in Eiras et al. [29] and Ritchie et al. [30].

**Table 1:** Sound-GAT bill of materials.

Item	Manufacturer/Supplier	Cost (US$) Per Unit
Arduino Pro Mini 3.3 V	Sparkfun/eBay	1.85
Speaker	PUI Audio/DigiKey	6.20
Capacitor	DigiKey	0.75
Battery Case w/ Switch	eBay	0.75
Photoresistor	DigiKey	0.40
Sound Lure total not incl. batteries		**9.95**
3x AA batteries	Varta/Element14	0.99 (0.33 each)
Sound Lure total incl. batteries		**10.96**
Gravid *Aedes* Trap	Biogents AG	13.63
Total Trap Purchasing Cost		**$24.59**

### Latin squares in field

Field studies were performed in the urban suburbs of Cairns North and Bungalow located in Cairns, Queensland, Australia known to have populations of *Ae*. *aegypti* [31]. All private homeowners gave permission and granted access for the study to be conducted on their property. A three x three Latin square (Sound-GAT, unbaited GAT and unbaited BGS) was randomly established and replicated across five (n = 5) housing blocks split across the two suburbs. This study design resulted in 15 trapping locations during which 5 of each trap type were deployed per replicate (*n* = 6). Each GAT was set with a single sound lure placed on the centre of the trap mesh inside the GAT head and programmed to playback a flight tone of 484 Hz at two-minute on/off intervals resulting in 16 operational hours per day (12 hr daylight + 4 hr twilight playback). An interval playback strategy was incorporated to save power and observations of male acclimation and decreased response during continuous playback (data not shown). Traps were serviced and rotated weekly and the entire Latin square design replicated once during the six-week sampling period from 8 February to 23 March 2017 for a total of 30 observations per trap type. During sampling, individual traps were placed within close proximity of a single residence (<5 m), sheltered from direct wind, sunlight and rain. All GATs were treated with a deltamethrin-based surface spray (Mortein Barrier Outdoor Surface Spray, Australia) and baited with a hay infusion (3 g hay per 3 L) at the beginning of each Latin square. BGS traps were unbaited (no CO_2_ or BG-Lure) and operated continuously. The decision to trap without the BG-lure or CO_2_ was based on previous reports indicating their addition at substantial costs does not significantly increase male capture rates over unbaited BGS traps [32, 33]. All *Ae*. *aegypti* sampled were identified and sex determined. The traps and methodology used did not impact any protected species.

### Statistical analysis

The effect of treatment (trap type), trap location (house), and trap week on the number of male and female *Ae*. *aegypti* captured was analyzed by three-way analysis of variance (SAS Institute 2001). The Ryan-Einot-Gabriel-Welsh multiple range test was used to separate mean differences and significant differences are based on *P<* 0.05. Capture data were square root-transformed before analysis, but actual numbers are shown in text. Statistical analyses were performed using IBM SPSS® software (IBM Corporation, Armonk, NY USA).

## Results

### Latin square field trials

**Fig 2:**
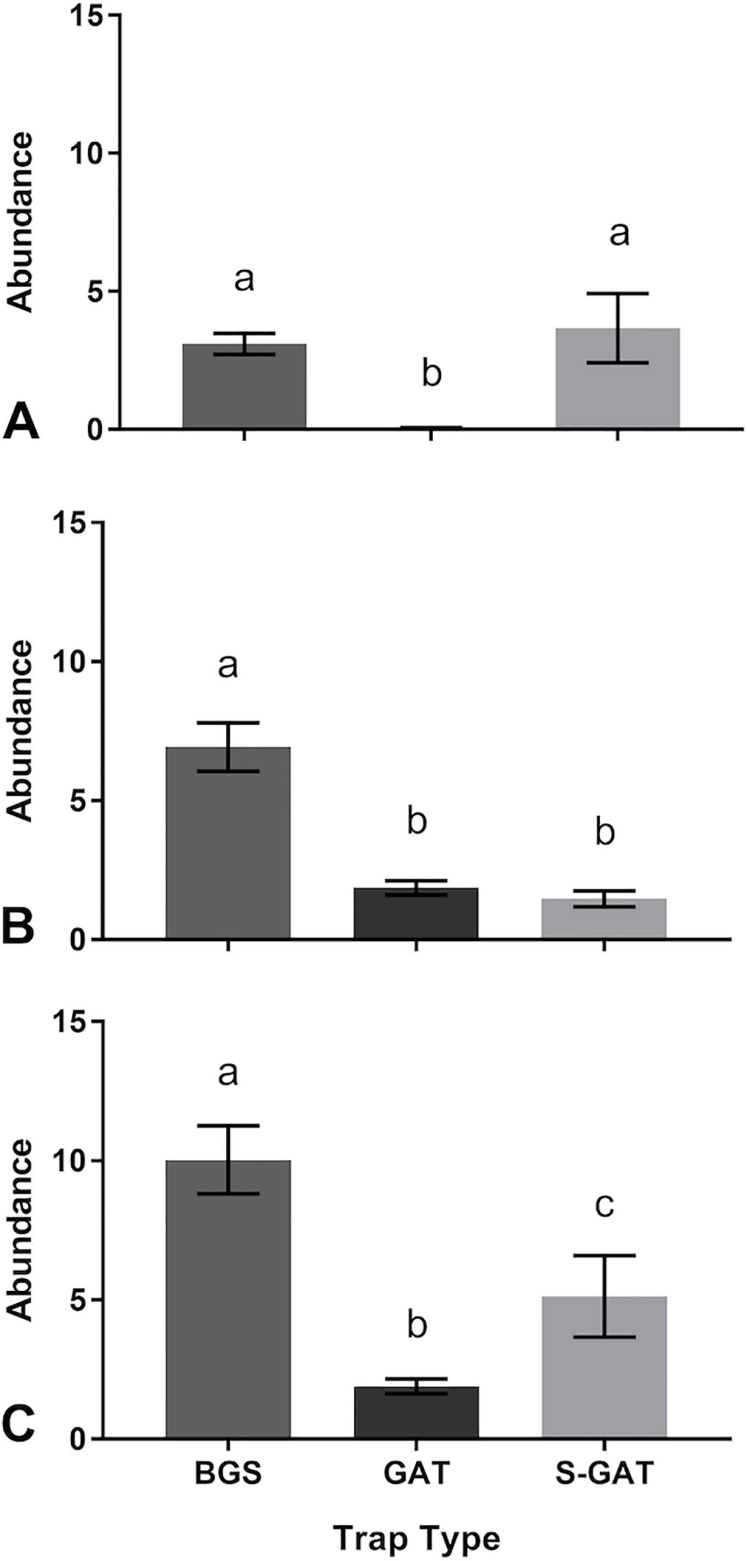
Male and female capture in biogents sentinel traps and Sound-GATs. **(A)** Mean weekly catches (± S. E.) of male *Ae*. *aegypti* caught by BGS, GAT and sound-baited GAT (S-GAT) traps. **(B)** Mean weekly catches (± S. E.) of female *Aedes aegypti* caught by BGS, GAT and sound-baited GAT (S-GAT) traps. **(C)** Mean weekly (± S. E.) *Aedes aegypti* trap totals (males and females) in BGS, GAT and sound-baited GAT (S-GAT) traps. Labels indicate significant groupings (*P* < 0.05, Factorial ANOVA, *n* = 30).

Mean male capture rates were not significantly different between the BGS and Sound-GAT traps (Fig 2A). In contrast, unbaited (no sound lure) GATs captured significantly fewer males than the Sound-GAT and BGS (Fig 2A; *F* = 39.2, df = 2, *P* < 0.001, *n* = 30). Overall, the weekly male capture ratio between the Sound-GAT and BGS was 1.18: 1. The BGS, GAT and Sound-GAT averaged 3.2±0.38, 0.03±0.03, and 3.67±1.25 males per week, respectively. Location (house) was found to be significant (*F* = 2.32, df = 14, *P* = 0.018), indicating particular houses being hotspots, but no interaction was found between location and trap type revealing location did not bias trap outcome. In this case, the means of one pair of houses was found to be significantly different across all five Latin squares. Sample week was found to be not significant (*F* = 0.43, df = 5, *P* = 0.73).

Female capture rates were significantly higher in BGS traps than in other trap types (Fig 2B; *F* = 39.6, df = 2, *P* < 0.001, *n* = 30), whereas female capture rates in the GAT were not significantly different to that of the Sound-GAT, importantly indicating no repellency effect of the sound lure on females. Overall, the weekly female capture ratio between the GAT (Sound-GAT and unbaited GAT) and BGS traps was 0.24: 1. Overall, the BGS, GAT, and Sound-GAT averaged 6.9±0.88, 1.9±0.28, and 1.5±0.29 females per week, respectively. Similar to male captures, location was found to be significant (*F* = 2.32, df = 14, *P* = 0.02), and again the means of a single pair of houses was found to be different. No interaction between location and trap type was observed and sample week was found to be not significant (*F* = 0.75, df = 5, *P* = 0.53).

Weekly trap totals (males and females) were highest in BGS traps (Fig 2C; *F* = 25.6, df = 2, *P* < 0.001, *n* = 30), followed by Sound-GATs, and lastly by GATs. Weekly collection ratios were 2.7: 1 between the BGS and Sound-GAT and 2.8: 1 between the Sound-GAT and unbaited GAT. Overall, the BGS, GAT, and Sound-GAT averaged a total of 10.03±1.38, 1.9±0.18, and 5.13±1.45 *Ae*. *aegypti* per week, respectively. Location was again found to be significant (*F* = 2.43, df = 14, *P* = 0.01, with the same single pair of houses having significantly different means as observed for female capture. No interaction between location and trap type was observed and sample week was found to be not significant (*F* = 0.73, df = 5, *P* = 0.55).

## Discussion

As male-based *Aedes* rear and release programs become more efficient and expand operations, they will require improved monitoring tools for cheap and accurate release tracking. Thus, the objective of this study was to make available an operationally affordable and practical version of the previously reported Sound-GAT [19] for effective male monitoring. The results demonstrate significant steps in achieving this goal by the development of an economical, long-lasting, and highly programmable microcontroller-based sound lure. GATs baited with this lure captured just as many males as the current “gold-standard” BGS trap and at significant cost savings. Thus, for the purposes of male monitoring, GATs baited with the newly developed lures represent extremely good value ($24.59 vs. $219.81 USD for the BGS, Table 2) for rear and release programs with the added benefit of being effective monitoring devices for gravid females and consequently arbovirus activity. Further cost savings could be found in scaled-up production of the sound lure with custom printed circuit boards, dramatically reducing per unit costs. These cost savings and simplified logistics enable researchers to increase trap coverage and would be particularly useful for monitoring male *Aedes* mosquito releases in developing countries where main power (household) is not easily accessed.

**Table 2:** Surveillance device cost (US$) comparison.

Item	Biogents Sentinel	Sound-GAT
Trap Cost w/o Battery (US$)	$197.00 (Bioquip Products, USA)	$23.60
Active current (mA)	280	5.8
Active Power (mW)	3360	29
Battery Cost per Trap (US$)	$22.81 (10 Ah, 12V; (Universal Power Group Inc., USA)	$0.99 (3 x AA)
Battery Capacity (mA-h)	10000	1800–2600
Active Battery Life (weeks)	0.21 (36 hr)	4.3–6.3 (30–44 days)
**Total Trap Cost**	**$219.81**	**$24.59**

In addition to cost savings, the power savings of the presented lure is perhaps its greatest attribute over the BGS. The power draw of the Sound-GAT is substantially less than the BGS resulting in enormous savings on battery costs and a greatly decreased risk of battery theft. While the BGS can require regular maintenance and battery replacement, field-deployed Sound-GATs would only require battery changes every 4–6 weeks based on predicted battery life. If set to also collect females, then the battery change could occur during trap maintenance to change infusion and reapply pesticides when needed [30]. A benefit on the long battery life is that the device does not necessitate more servicing than needed for the trap itself, which is commonly serviced weekly, biweekly or monthly depending on operator needs. Of note, if the small LED light above the reset button on the Arduino board is removed, battery life can be extended to 4.9–7.1 weeks (34–50 days) as the active current drops to 4.8 mA. This is a great improvement over the 24–48 h battery life of a typical 12 V (10–20 AH) rechargeable used to power a BGS trap. The schematic (Fig 1) for this device is included in this publication for low-cost, local fabrication of Sound-GATs in developing countries. The programming code is available upon request (correspondence to BJ Johnson) to further ease adoption of this methodology.

Finally, as *Aedes* rear and release programs expand their efforts to control vector populations (e.g. Oxitec [13], Verily Debug Project [11], MosquitoMate [12], IAEA [9, 14]), improved monitoring tools are needed for cheap and accurate male-release tracking. Although the Sound-GAT allows for accurate, low-cost monitoring of male *Ae*. *aegypti*, such a system still requires laborious and expensive physical collection and identification of captured mosquitoes. Ideally, to maximize surveillance effort and minimize costs, efforts should be made to combine the “sound lure” concept with an Internet of Things-based sentinel detection and reporting system, eliminating continuous visits to individual traps and providing real-time information about the spatio-temporal population dynamics of the target population before and after release. Such systems would provide more specific and accurate data allowing operators to better target “hotspots”, increasing efficacy while reducing labor costs and logistics. Sentinel systems would also be useful in identifying cryptic *Aedes* infestations that often go unnoticed during routine surveillance efforts, as well as detect and enable rapid response to new, post-treatment infestations. Steps in this direction have been made with recent advances in optical sensors, mostly infrared-based light arrays, which detect small variations in the light captured by the phototransistors as the insect crosses the beam during entry into the trap [34–36]. These variations are recorded as an audio signal, which can then be used to differentiate species and males and females based on their unique wing-beat frequencies. Although field reports for mosquitoes are lacking, laboratory assessments have shown promise, particularly with advanced machine-learning classification algorithms. Further refinement of components, such as the presented sound lure, will help make these systems viable for wide-scale surveillance.

### Conclusion

The expansion of male-based rear and release *Aedes* control operations have highlighted a critical need for economical, male-focused surveillance devices for accurate release tracking. We herein developed a low-cost, microcontroller-based sound lure that produced male *Ae*. *aegypti* mosquito catch rates comparable to those of the BGS when placed inside the low-cost, passive GAT. Hence, for the purposes of male monitoring, sound-GATs represent extremely good value for rear and release programs. Further, the significantly reduced power consumption of the sound lures themselves make solar power a much more viable option, ideally enabling power to be allocated to telemetry and other future ‘smart’ trap features to allow for the remote detection and transmission of trap data. Such traps would result in a dramatic drop in labor cost, and a significant improvement in latency of detection. Thus, the sound-enhanced trap is a key stepping stone on the path to an automated monitoring system for male-based suppression programs and surveillance.

## Supporting information

S1 Table Latin square collection summary for male and female *Aedes aegypti*. (XLSX)
